# Pittcon Abstracts (1991)

**DOI:** 10.1155/S1463924691000147

**Published:** 1991


Journal of Automatic Chemistry, Vol. 13, No. 2 (March–April 1991), pp. 59–79

### Pittcon Abstracts (1991)

### Speciation studies of selenium and arsenic by reversed-phase high-performance liquid chromatography, employing hydride generation and inductively coupled plasma mass spectrometric detection

*W. Charles Story, E. Hywel Evans, Douglas T. Heitkemper and Joseph A. Caruso (Department of Chemistry, University of Cincinnati, Mail Loc. 172, Cincinnati, OH 45221-0172; National Forensic Chemistry Center, U.S. Food and Drug Administration, 1141 Central Parkway, Cincinnati, OH 45202)*

Different forms of metals and metalloids have varying degrees of toxicity in biological systems. Because of this, there has been an increase in the research focused on the speciation of metals at trace levels. Plasma mass spectrometric detection of hydride species has been established as a viable technique for the determination of these species at trace levels. One problem with the determination of arsenic by plasma mass spectrometry is the possibility of interference from the formation of $ArCl^+$. Since many naturally occurring samples contain chloride, direct nebulization of the sample will introduce chloride to the plasma. A possible solution to this problem is the use of hydride generation to convert the arsenic to a volatile form coupled with separation from the chloride using a membrane gas liquid separator of the type used previously.

The effectiveness of using hydride generation and the membrane separator in reducing the $ArCl^+$ interference on the detection of arsenic was presented. A high-performance liquid chromatographic separation of several arsenic and selenium species demonstrate the simultaneous detection and speciation of these two elements using hydride generation to increase the sensitivity. Figures of merit were discussed, along with the pertinent experimental details.

### Addition of molecular gases to Ar gas flow for the reduction of polyatomic ion interferences on arsenic and selenium in inductively coupled plasma mass spectrometry

*Jiansheng Wang, E. Hywel Evans and Joseph A. Caruso (Department of Chemistry, M. L. 172, University of Cincinnati, OH 45221)*

Due to its sensitivity and multi-element capability, Inductively Coupled Plasma Mass Spectrometry (ICP-MS) has developed into a very powerful technique for the determination of trace elements. However, the choice of hydrochloric acid for sample dissolution, or a high concentration of chloride in sample, makes it almost impossible to determine arsenic or selenium because of interferences caused by $^{75}ArCl^+$ on $^{75}As^+$, $^{76}ArAr^+$ on $^{76}Se^+$, $^{77}ArCl^+$ on $^{77}Se^+$ and $^{78}ArAr^+$ on $^{78}Se$. Different methods, such as co-precipitation of chlorides, chromatographic de-salting, alternative sample preparation procedures and addition of molecular gases to the central channel Ar gas flow have been used to overcome these polyatomic interferences.

In this study, the effects of addition of molecular gases such as nitrogen to the central channel, the coolant and the auxiliary flows have been investigated. A standard reference material and real fusion samples containing a high concentration of HCL have been studied. The effects of the addition of molecular gases to Ar flows on polyatomic arsenic and selenium were shown.

### Vapour generation techniques to measure arsenic by atomic fluorescence

*P. B. Stockwell (P. S. Analytical Ltd, B4 Chaucer Business Park, Kemsing, Sevenoaks, Kent TN15 6QY, UK; L. C. Ebdon and Warren Corns, Polytechnic South West, Drake Circus, Plymouth PL4 8AA, UK); and K. C. Thompson (Yorkshire Water Enterprises Ltd, Charlotte Road, Sheffield S2 4EQ, UK)*

Vapour generation techniques have proved immensely valuable when measuring environmentally important elements at the levels present by existing techniques. Sensitivity improvements of 500 times are not uncommon for such measurements when linked to Atomic Absorption and Inductively Coupled Plasma Spectroscopy. Recently Stockwell *et al.* have described additional benefits of coupling such simple preparative techniques to atomic fluoresence. A specific system designed for mercury analysis provides simple instrumental systems which can measure reliably down to few parts per trillion.

The determination of arsenic by hydride generation atomic fluorescence spectrometry using both non-dispersive and dispersive systems is well established. However, to date, there is no commercially available instrumentation for this technique, this is largely due to the lack of reliable high intensity sources.

The paper described the use of a boosted discharge hollow cathode lamp to excite arsenic atoms obtained from various designs of atom cells. The relative advantages and disadvantages of using dispersive and non-dispersive systems will also be discussed.

Applications of environmental interest were described with validation using a wide range of certified reference materials at different levels, a fully atomated system was also described in detail.

### Determination of tin using hydride AAS including flow injection analysis (FIA) and trapping in a graphite furnace

*Zhang Li, Susan McIntosh, Glen Carnrick and Walter Slavin (The Perkin-Elmer Corporation, 761 Main Avenue, Norwalk, CT 06859-0237)*

The determination of tin at ultratrace levels is very important because the metal has an important environmental role even at very low levels. There is little information in the literature on the tin determination as the hydride, although it has been reported as difficult because the hydride is reported to be unstable.

The hydride method provides the opportunity to measure very low concentrations of Sn and, at the same time, to separate the element from potential interferences. The simplicity and convenience of flow injection analysis (FIA) has been used to explore the parameters of the Sn determination as the hydride.

Still greater concentration sensitivity was found by trapping the Sn hydride in a warm graphite furnace, and then atomizing the Sn using conventional STPF conditions for Sn. The characteristic mass for Sn in pg by this procedure was the same as when solutions containing Sn were analysed in the furnace but large volumes of sample can be analysed. Interferences were small using furnace trapping.

The detection limit was 7 ng/l for a 10-ml sample, probably the best instrumental, non-laser, detection limit ever reported for Sn. The analytical precision was 3 to 5% RSD at 1 ng of Sn. Inter-element interferences were investigated. The recommended method has been applied to the analysis of Steel, River Sediment and Orchard Leaves and Bovine Liver Standard Reference Materials, SRM's and this data was presented.

---

### The rapid determination of traces of selenium and tellurium in copper electrolytes

*John Bozic, R. L Ethier, J. M. Balleny (INCO Limited, Central Process Technology, Copper Cliff, Ontario, P0M 1N0, Canada)*

INCO Limited is a major producer of high quality copper cathodes, using both electrorefining and electrowinning processes at its Copper Cliff Copper Refinery.

For process control purposes, and to ensure product quality, the rapid determination of Se and Te levels in copper electrolytes down to sub parts-per-million levels is very important. Traditional procedures have relied upon separation of the analytes by distillation, co-precipitation, solvent extraction, etc. prior to spectrophotometric measurement. Such procedures are slow and labour-intensive. The authors investigated the capability of a number of modern instrumental analysis techniques to determine Se and Te in the difficult matrix containing, for example, 200 g/l $H_2SO_4$, 100 g/l Cu, and 10 g/l Ni, Co. Direct ICP-AES and DCP-AES procedures lacked sufficient sensitivity and suffered from major inter-element interferences. Flame AAS with deuterium arc background correction was also insensitive.

Anodic stripping voltammetry was found to be impractical due to interferences from organic additives present in the electrolytes.

Electrothermal Zeeman AAS and polarized flame Zeeman AAS were both found to be capable of providing precise and accurate Se and Te analyses with minimal sample treatment. For routine analysis the latter procedure is favoured.

Te is measured using an air-acetylene flame, while an argon-hydrogen-air flame is used for Se. Matrix matched standards are essential. Details of analytical procedures and comparisons between methods were presented and applications of polarized flame Zeeman AAS to other difficult matrices were outlined.

---

### Determination of hydride-forming elements in metals by flow injection atomic absorption spectrometry with on-line matrix isolation

*S. G. Offley and N. J. Seare (Department of Chemistry, University of Technology, Loughborough, Leicestershire LE11 3TU, UK); J. F. Tyson (Department of Chemistry, University of Massachusetts, Amherst, MA 01003); and H.A.B. Kibble (Philips Scientific, York Street, Cambridge CB1 2PX, UK)*

Although the determination of elements such as As and Se by the generation of the volatile hydride with subsequent atomization in a tube furnace is a mature technique, there are a number of limitations to the procedure. These arise principally from the nature of the matrix components. Determinations of trace elements in transition metals are particularly difficult due to the involvement of the matrix element in a competing reaction with the reductant (borohydride) and the subsequent reaction of the determinands with the reaction products.

This work described the application of a continuous flow manifold which removes the matrix element from the analyte solution by retention on a cation exchange column. The resulting solution flows through the loop of a flow injection (FI) valve and a discrete volume is injected into a FI manifold in which it is merged with streams of acid, borohydride and argon prior to separation of the vapor in a glass U-tube device. This novel flow injection manifold design allows independent optimization of the matrix isolation and hydride generation chemistries. The various operations may be fully automated, including that of column regeneration.

Optimization of the FI manifold for the determination of Se was carried out using an alternating variable method. Optimum sensitivity was determined to be dependent not only on reagent concentration but also on the total reagent flow rate in the manifold. Optimization of sensitivity for interference tolerance for both copper and nickel was achieved through maximizing the total reagent flow rate (15 ml $min^{-1}$). Reducing the injection volume was observed to increase the interference tolerance.

The accurate analysis of two standard reference copper materials (BAM 361 and NIST 454) was carried out, containing 30 and 479 mg $kg^{-1}$ Se(IV) respectively. A throughput of 17 $h^{-1}$ was possible with column regeneration after each sample (triplicate injections of each sample). A characteristic concentration of 1·0 ppb was achieved with an RSD of 1·5% (10 ppb, N = 12).

The FI manifold has also been optimized for the determination of As(III). Performance was observed to be significantly affected by the condition of the silica tube-in-flame atomizer. Conditioning procedures were investigated. Investigation was made into the interference tolerance of the system for Ni, Cu, Co(II), Sn(II), Sb(III) and Se(IV). Incorporation of an in-line membrane filter was observed to eliminate interference memory effects associated with high concentrations of Cu and Ni and improve the precision.

---

### Determination of As, Sb, and Se with electrothermal vaporization into a helium microwave-induced plasma

*Jorge Alvarado and Jon W. Carnahan (Department of Chemistry, Northern Illinois University, DeKalb, IL 60115)*

Electrothermal vaporization (ETV) has been shown to be an excellent method for the introduction of aqueous samples into helium microwave-induced plasmas (He-MIP). In a comparison study of ETV and ultrasonic nebulization (USN), Wu and Carnahan have shown the ETV to exhibit superior Cl and Br detection limits by two orders of magnitude. The authors have shown similar behavior for the analysis of As, Sb, and Se.

For this study, the sample introduction system consists of a carbon cup type electrothermal vaporizer coated with tantalum in a glass chamber. A secondary pyrex vapor restriction device is used to enhance the efficiency of sample transport to the plasma.

Detection limits obtained with ETV (15 µl sample) and USN, respectively, were: SB, 0·008 mg/l and 0·098 mg/l at 259·805 nm; Se, 0·122 mg/l and 13·2 mg/l at 203·985 nm; and As, 0·015 mg/l and 0·750 mg/l at 234·984 nm. To obtain the best detection limits nickel nitrate and lead nitrate were used as matrix modifiers. Linear responses of three orders of magnitude were obtained for each element.

---

### Simultaneous ICP-AES determination of As, Sb, and Se by hydride-generation sample introduction

*L. J. Prell, J. D. Lindner, V. A. Letourneau, and D. E. Dobb (Lockheed Engineering and Sciences Company, 1050 E. Flamingo Rd., Las Vegas, NV 89119), and T. A. Hinners (US EPA, Environmental Monitoring Systems Laboratory, 944 E. Harmon, Las Vegas, NV 89119)*

The multi-element capability of ICP-AES has not been realized for low level As, Sb, and Se determinations. Pneumatic nebulization alone does not provide enough sensitivity for these elements. However, hydride-generation (HG) sample introduction greatly improves ICP-AES sensitivity for these elements. To optimize this sensitivity, Hg sample introduction requires conversion of analyte species to their most efficient hydride-forming oxidation states. Presently, HG techniques for As and Sb require the addition of KI to form the +3 oxidation state. However, KI reduces Se to its elemental form, which does not form hydrides. Selenium's most efficient hydride-forming oxidation state is +4, which is attained by high concentrations (20%) of HCL. Since the conditions for HG differ between Se and the As/Sb pair, analyses of these elements were historically performed separately. However, there is no need to separate the analyses once the hydrides are formed. Thus, a continuous-flow hydride generator, which splits the sample flow into two reaction streams, was used for simultaneous determination of all three elements. One reaction stream (3·0 ml/min) was heated to 70 °C and mixed directly with 0·28 ml/min of 0·1% $NaBH_4$. This allowed the selenium hydride to form. The other reaction stream (3·0 ml/min) was heated to 70 °C and mixed with 0·42 ml/min of 1% KI before it was mixed with 0·28 ml/min of 0·1% $NaBH_4$. This allowed the As and Sb to form hydrides. The streams were then combined and introduced into a Hildebrand-grid nebulizer and water-jacketed Scott Spray chamber chilled to 1 °C. This combination served as a highly efficient gas–liquid separator. The argon carrier gas pressure was 10 psi (100 ml/min), which was too low to cause nebulization of the aqueous phase that is laden with salts. With the Hildebrand-grid nebulizer, a significant mist is not observed unless the pressure is greater than 40 psi. The analytical figures of merit were evaluated using an ARL 3560 mini-torch ICP-AES with synthetic solutions and digests of NIST Fly Ash and NIST River Sediment. The relative standard deviation was 1·5% for As and Sb, and 5% for Se. The detection limits (DLs) were 0·8 µg/l for As and Sb and 5 µg/l for Se. The Se DL was not affected by the compromise HG conditions used for the other analytes. The linear range extended to 100 µg/l for all three elements. Above the 100 µg/l level, pneumatic nebulization provides sufficient sensitivity. The developed analytical procedure is a candidate method for approval by the US EPA.

Notice: Although the research described in this article had been supported by the United States Environmental

Protection Agency, it has not been subject to Agency review and therefore does not necessarily reflect the view of the Agency and no official endorsement should be inferred. Mention of trade names or commercial products does not constitute endorsement or recommendation for use.

---

### A practical approach to automated ultra-low level mercury determinations

*Doug Shrader and Johnathan Moffett (Varian Optical Spectroscopy Instruments, 201 Hansen Court, Suite 108, Wood Dale, IL 60191)*

A continuous flow cold vapor generator (VGA-76) is capable of routine detection limits of 0·2 μg/l by atomic absorption. Increasingly, legislation around the world is specifying mercury levels to be measured at sub-ppb levels. European regulations require measurements to be made at 0·05 μg/l. This implies that a system should be able to measure to an order of magnitude lower than 0·2 μg.

For a number of reasons, atomic absorption is the logical choice to measure at these ultra-low levels. These include its excellent sensitivity, adaptation to routine measurement and cost-effectiveness.

The ease with which gold can form an amalgam with mercury suggests a method to improve sensitivity. A gold collecting surface can be inserted into the mercury vapor flow. After a suitable collection time, the adsorbed mercury can be quickly released by electrical heating of the gold collector. This gives an enhanced transient mercury signal. This study shows that the enhancement is directly proportional to collection time. Calibration curves are linear up to a mass of mercury equivalent to 100 ng and absorbances of about 1·0.

In routine accurate measurements of sub-ppb levels of mercury, several analytical challenges present themselves. For instance, reagent contamination alone is frequently greater than 0·05 μg/l. Technical problems and their resolution were discussed. These include minimization of extraneous mercury being trapped on the collector, interaction with an automatic sampler and various atomization cell designs.

---

### The determination of tin in complex environmental samples by hydride generation atomic absorption spectroscopy

*Cindy Beach and Doug Shrader (Varian Optical Spectroscopy Instruments, 201 Hansen Court, Wood Dale, IL 60191)*

Hydride generation AA techniques are used routinely for the determination of As, Se, and Sb in environmental samples. Advantages include high sensitivity, simplicity, and relative freedom from interferences. Tin can also be determined by hydride generation, however, current methodology suffers from severe difficulties. These include pH dependency, potential contamination problems poor standard stability, and severe metal interferences.

This paper described the development of a relatively simple tin hydride generation AA method that eliminates these problems. In this study difficult environmental sample were analysed for tin by continuous flow hydride generation. Continuous flow designs greatly reduce analysis time as well as improve precision and allow for automation. Various masking or complexing agents have been found to be extremely beneficial, not only in the elimination of metal interferences as reported by others, but also in significant improvements in sensitivity, precision and stability. A surprising aspect of the method was little pH dependency. Standards and samples contained 1% $HNO_3$ at a pH of <10. A simple $HNO_3$/L-cysteine method was investigated in the analysis of digested complex environmental samples.

---

### An automated laboratory for analysis and control of aluminum surface preparation processes

*James W. Peterson, Bruce R. Davis, Harvey E. Siedenburg and Donna M. Vanbramer (Quality Assurance Research and Development, Boeing Commercial Airplane Group, P.O. Box 3707, M/S 9R-58, Seattle, WA 98124-2207)*

Surface cleaning and anti-corrosion treatments for aluminum parts are critical processes in the aerospace industry. An automated laboratory for analysing and controlling these tank-based processes has been designed and installed by Boeing Commercial Airplanes in its Sheet Metal Center in Auburn, Washington. Using inductively coupled plasma spectometry for metals analysis, ion chromatography for anion and triethanol amine measurement, and automated titrations for pH and oxidation state determinations, the laboratory monitors all performance-related chemical parameters of the 44 tanks involved in the factory's 15 process sequences. Each analytical instrument is microprocessor controlled and performs a variety of programmed analytical methods without operator intervention. Sample collection, preparation, and delivery is performed by a system of computer-controlled valves and pumps from a continuously refreshed source for each process tank.

The activities of the instruments and sampling system are orchestrated by a MicroVAX computer using Boeing-developed software. Operating from a user-defined test schedule, the MicroVAX supervises the simultaneous operation of the sampling system and instruments, transmitting commands to each, and receiving the results of all analyses. From the analytical data, the status of each tank is compiled and transmitted via a local area network to a VAX cluster which coordinates all factory activities. The VAX cluster is responsible for archiving the data, providing statistical analyses, and calculating control actions to maintain the process tanks at their optimum conditions. The control actions are executed via programmable logic controllers on the factory floor.

---

### Automated flow-through microwave digestion for on-line sample preparation and inductively coupled plasma spectrometry

*Ramon M. Barnes and Laura J. Martines (University of Massachusetts, Department of Chemistry, Lederle GRC Towers, Amherst, MA 01003-0035)*

Innovative sample preparation for accurate elemental determinations, especially with inductively coupled plasma spectrometry, has been stimulated by the availability of commercial microwave digestion devices that provide benefits compared to conventional open beaker, hot plate digestions. Closed and open vessel microwave oven digestions are effective techniques that improve precision and accuracy, reduce contamination, and increase sample throughput. One practical limitation results from the sealing of closed vessels and the insertion of vessels into the microwave oven. The combination of microwave digestion and flow techniques was first reported by Burguera *et al.*, and has subsequently been investigated by others. Continuous or stopped-flow online microwave digestion can increase sample throughput and extend sample handling automation.

A commercial laboratory microwave oven is available (Prolabo A300) with the capability to perform automated digestions by utilizing microprocessor control and a mechanical arm. The microwave energy in this oven is focused in a small cavity. In one approach to combine microwave digestion with sample flow techniques, the sample tubing is placed in the digestion cavity. As the sample and acid are pumped through the tubing inside the cavity, the microwave energy can heat the acid and controlled sample digestion can occur.

Several experimental considerations must be made for the practical development of this approach. The acids type and volume, use of flowing streams and stopped flow techniques, settling of solids from the reaction solution, control of reaction pressure, and formation of gas bubbles are some of these considerations. These effects and the analytical characteristics of flow-through microwave digestion techniques will be described. An expert system that helps to select the digestion parameters can be interfaced with the flow-through system to enhance automation of the digestion methods development.

---

### Sample preparation of oxide materials using the pulverizing, pelletizing and fusion method under routine conditions in fully automatic systems

*G. Hawickhorst (HERZOG Maschinenfabrik GmbH, P.O. Box 2329, D-4500 Osnabrück, Germany)*

Almost 15 years ago the first installations of fully automatic sample preparation systems based on pulverizing and pelletizing had been established in the cement industry. The first systems based on the fusion technique have been installed during the last few years.

With improved speed, performance and reliability of the third generation of those systems, entry was made in steel plant-, mining- and other laboratories.

The advances in electronics and robotics gave the necessary tools to automate the fusion process. This process is much more sensitive to trouble-causing effects such as dust, vibration etc. than the pulverizing and pelletizing process.

The fused bead eliminates several negative effects in the sample (mineralogical, particle size, etc.), but on the other hand there is the danger of cracking of the glass disk due to internal stress.

Beside the analytical requirements these facts also have to be taken ito account when deciding which method to use for fully automatic on-line systems under routine conditions for process control.

With both systems there is enough experience available to judge in which field of application which system will promise the best success concerning analytical results, speed, lowest maintainance cost, and reliability.

Layouts of existing installations were shown and the latest experiences with installations in various industries were reported.

---

### The adaptation of FIA for the determination of phosphate in phosphate ore

*Franklin E. Patrick and John E. Gibson (IMC Fertilizer Incorporated, P.O. Box 876, Bartow, FL 33830)*

IMC Fertilizer is the world's largest private producer of phosphate rock. As such, the capability for rapid and accurate determinations of phosphate in phosphatic ore is crucial. In the authors' lab, the following criteria have been designated as necessary to provide this capability:

(1) Throughput of at least 60 samples/hour.

(2) A range from 10 to 1500 mg $P_2O_5$/l.

(3) Relative standard deviation of less than 0·5% at the upper end of the range.

These criteria reduced the number of possible analysers to a select few. The Tecator 5010 FIA Analyzer was evaluated and was found acceptable.

The Tecator 5010 Analyzer is an automated flow injection instrument interfaced with a personal computer using the manufacturer provided software, SuperFlow II. The standard molybdo-vanadate procedure as described in the *AOAC Officiall Methods of Analysis* was adapted to the instrument. This paper reported on the manifold configuration, flow rates, and other considerations necessary to run this method on the analyser and meet the stringent analytical requirements. Calibration is accomplished by analysing phosphate rock standards covering the range encountered in digested rock samples. Data from these standards are processed using a polynomial curve fitting routine to obtain a calibration curve that accurately fits the non-linear absorbance versus concentration relationship.

Data from the Tecator Analyzer compared favorably with data from three different instruments: an air-segmented continuous flow analyser, a discrete volume automated batch analyser, and an ICP. The throughput of the system is 90 samples/hour. A standard deviation of 4.0 mg $P_2O_5$/l was established for samples at the higher end of the cencentration range.

The module design of the analyser was also an important consideration. There are currently three analysers at two separate locations and there are plans to add another one at a third location. The flexibility offered by the design allows components to be swapped if needed to reduce down time.

---

### A practical system approach to NIR industrial process analysis

*Barry Read (Bran + Luebbe Analyzing Technologies, Inc, 103 Fairview Park Drive, Elmsford, NY 10523)*

Successful implementation of spectroscopic analysis in the process control environment requires knowledge of the physical and chemical properties of the constituent to be analysed, as well as its corresponding matrix.

In addition to the fundamental spectrochemistry, the proper sensor must be selected for the application to ensure accurate and reliable data collection. Next, sample handling techniques must be optimized for the sensor and the individual process environment.

Once these factors have been solved, data acquisition, process equipment interface and integration of the existing automation processes is required before the system can be truly considered as an in-line sensor.

The implementation strategy used in placing near infrared sensors into the process environment were discussed with some real world examples of these projects.

---

### Flexible sample and data handling systems for the determination of total nitrogen sulphur

*John S. Crnko, Dewey Smith and Mike Martin (Antek Instruments, Inc. 300 Bammel Westfield Rd., Houston, TX 77090)*

Sampling and analytical techniques for the new total nitrogen and total sulphur analyser were presented. Descriptions of the various equipment used to eliminate or minimize sample preparation for many different sample types were included. New data handling systems with customized software are utilized in a stand alone integration system, a personal computer or as an on-board function of the Antek Analyzers. All of these systems can be used in both manual and autosampler modes.

The ability to analyse solids, liquids, and gases by using a single pyrotube configuration is highlighted. Also a new more efficient inlet oven for solid materials will be described.

Data generated using a new autosampler for solids materials and a new gas sample injection system are included.

Examples of operational characteristics are illustrated to show peak integration flexibility, data archival, report formats, quality assurance measures and ease of use.

All system descriptions are accompanied by application data including measurement at ppb levels for both nitrogen and sulphur. Materials analysed include both organic and inorganic matrices such as petroleum products, polyolefins, petrochemicals, and agricutural products.

---

### The use of automated gravimetric techniques for enhanced analytical precision

*Brian G. Lightbody and Sally D. Dowling (Zymark Corporation, Hopkinton, MA 0178)*

The use of automated equipment for routine sample preparation is growing, however, it is important to maintain a high level of control to maintain assay precision. Recent developments in automated workstations have resulted in capability to use an on-board 4-place analytical balance to monitor the progress of the sample prep process. This paper discussed several techniques built into the automated instrumentation which use analytical balance results to help ensure the precision and integrity of the analytical process. Some examples are as follows:

(1) The use of concentrate weight to calculate and add a diluent during automated dilutions.

(2) The use of sample weight to calculate and add a proportional amount of solvent.

(3) The use of sample weight to calculate and add a proportional amount of internal standard.

(4) The monitoring of the weight of all reagent additions to ensure adequate system performance.

---

### Continuous monitoring of personal exposure

*Eirik Nordheim and Helge Nes* (Aluminiumindustriens Miljøsekretariat, Sigurd Syrs gate 4, N-0273 Oslo 2, Norway; *Elkem Aluminium Mosjøen, P.O. Box 348, N-8651 Mosjøen, Norway)*

Industrial hygiene monitoring of personal exposure is traditionally carried out with sampling equipment like filters, impinger or adsorbent tube connected to a sampling pump or alternatively spot sample with hand-held adsorbent tubes. The latter type gives a crude estimate of short-time exposure while the first type gives an average exposure over the time period measured. In addition the result of the measurement is quite often not available for some time due to time-consuming laboratory analysis. For some specific applications direct reading instruments or dosimeters are now available, but most of them do still have a fairly limited application due to lack of continuous recording capacity.

Recently, lightweight dataloggers which are battery operated and with large datastorage capacity have started appearing on the market. These are flexible instruments with several input ports and capable of recording signals from a wide variety of sensors.

In the Norwegian aluminium industry the authors have over the last years tested out the use of a specific datalogger with 42 K storage capacity and 16 channels. This has been used together with sensors for CO, $SO_2$, respirable dust, static magnetic fields, body vibration, body temperature, pulse, skin temperature etc.

For most of these factors recordings have been made at one second intervals over an 8-hour shift period, giving a continuous record of exposure during a work period. The datalogger will thus give information about average exposure over a selected time period and, since time is also recorded continuously, be able to show short-time high exposure peaks with exact time when these occurred. For the purpose of health related monitoring these are often of more interest than the 8-hour average exposure.

The datalogger is connected to a PC after use for transfer of data, complete printout and graphical presentation. This can be done immediately after the measurement is finished. The operator who has carried the equipment, may be given a graph showing his exposure that day while he is still at work.

This lecture gave a complete description to the equipment, together with examples from actual monitoring in aluminium smelters, and also discussed the advantages of this type of monitoring in relation to employee health.

---

### Laboratory automation occurring at municipal wastewater treatment plants

*Gregory A. Zelinka (Madison Metropolitan Sewerage District, 1610 Moorland Road, Madison, WI 53713)*

Because of operational, liability, or permit requirements, today's municipal wastewater treatment plants are required to analyse a variety of sample matrices for a complex set of chemical and biological parameters. These matrices include: soil, plant tissue, ground water, surface water, and industrial wastes, as well as the more traditional municipal wastewater and sludges. The chemical parameters involve nutrients, metals, and organics, many existing at concentrations equal to or below current method detection limits. To meet these new and changing demands as well as keep up with the ever increasing sample load, it had become necessry for wastewater treatment plants to automate as much of the routine chemistry as possible. This paper discussed one of the ways Madison Metropolitan Sewerage District has attempted to automate its analytical workload.

One of the major areas intially examined was automating our solids and nitrogen procedures. Measurements for solids have been enhanced by coupling an electronic balance to a PC-based spreadsheet. This has reduced the tedium of recording weighings by hand, sped-up the calculations, and minimized the data handling errors. Nitrogen (NH3 & TKN) analyses have been automated by switching analytical methods from a Macro Kjeldahl method to a Semi-Automated method utilizing an Autoanalyzer. This has not only saved analytical time, but also energy and reagents as well.

A second area examined was the addition of autosamplers and timers to two of GCs, an IC, and an AA. This addition has allowed these instruments to operate unattended beyond regular working hours or over the weekend.

Data acquisition is being accomplished by dedicated PCs distributed throughout the lab and through the use of standardized data acquisition software. The authors are currently able to track the analog output from AAs, Autoanalyzer, IC, and GCs with a single vendor's software.

Data quantitation utilizes a series of specialized programs written in a compiled basic, which meets specific quantitation requirements and allows for custom report generation.

Quality assurance data is either automatically extracted and calculated (from the custom report format) or a manually entered into a daily QA report which flags control limit exceedances and facilitates monthly and quarterly report summaries.

---

### An automated, small-volume, block-digester procedure for the high-speed, simultaneous determination of total Kjeldahl nitrogen and total phosphorus in water samples

*Charles J. Patton (U.S. Geological Survey, National Water Quality Laboratory, 5293 Ward Road, Arvada, CO 80002)*

About 20 000 surface and ground water samples are analysed for total Kjeldahl nitrogen (TKN) and total phosphorus (TP) at the US Geological Survey National

Water Quality Laboratory (NWQL) each year. Recent estimates indicating that sample loads for TKN and TP will double by 1993 have spurred efforts to streamline all steps of these lengthy and labor intensive tests. Significant improvement in processing rates for pre- and post-digestion sample preparation, sample digestion, and determination of ammonium and orthophosphate ions in diluted digests have been achieved for these determinations relative to current NWQL procedures.

The NWQL currently uses two different digestion procedures – a Kjeldahl, block digestion procedure similar to US Environmental Protection Agency (EPA) method 351.2, and an acid/persulfate, autoclave digestion procedure, similar to EPA method 365.1 – for TKN and TP determinations, respectively. By determining TP in TKN digests, as per EPA method 365.4, sample preparation time is halved. Time required for the digestion step was reduced from four hours to one by halving sample and reagent volumes as per Jirka *et al.* Using syringe pump-based dispenser/diluter modules to automate pre- and post-digestion sample preparation permits these operations to be performed at rates of about 100 samples $hr^{-1}$, which is comparable to the rates at which ammonium and orthophosphate ions in diluted TKN and TP digests can be determined using third-generation continuous flow analysers.

In addition to increased production capacity, benefits afforded by this approach include decreased exposure of analysts to caustic and toxic materials, lower reagent and reagent preparation costs, and lower costs associated with proper disposal of caustic and toxic wastes resulting from these tests.

---

### Selecting a continuous flow analyser for the analysis of sludge and industrial waste effluents

*Shirley Arment and R. B. Roy (ALPKEM Corporation, 14625 S.E. 82nd Drive, Clackamas, OR 97015)*

Both air segmented (e.g. AAII and RFA) and nonsegmented (FIA) continuous flow analysers are available for the analysis of sludges and industrial waste effluents. In recent years the originally introduced CFA Technology, such as that of the AAII, have undergone much improvement both in mechanical designs and incorporation of computer capabilities.

For instance, the RFA (Rapid Flow Analysis) system was devloped to use glassware with reduced internal diameter, electronic timing of the segmentation gas, pecking sampler and 'gated' detector which needs no debubbling of air in the flowcell. Thus, the system provides faster throughput and low consumption of reagents, which in turn, reduced the cost of reagent and waste disposal.

FIA (Flow Injection Analysis) systems have similar features as the RFA except the FIA does not require air bubbles to separate samples. Both RFA and FIA have improved analytical performances in comparison to AAII systems. Unlike AAII systems, attainment of a steady state to analyse sample is not required in both RFA and FIA systems. Sample analysis rates well over 100 samples per hour are easily achieved.

The advantages and limitations of continuous flow analytical systems which are currently used in municipal and industrial laboratories were presented. In addition, performance data which will review design aspects of ALPKEM RFA systems, and, show applications demonstrating their various capabilities for environmental analysis were discussed.

---

### Ammonia and total Kjeldahl nitrogen determinations using fast distillations and gas diffusion

*Bernard Bubnis (Novatek, 10 West Rose Avenue, Oxford, OH 45056), and Jurgen Moller (Tecator AB, Hoganas, Sweden)*

Automated procedures for measuring ammonia and total Kjeldahl nitrogen (TKN) have been comparatively tested against a currently accepted EPA method.

Three test procedures for measuring ammonia and TKN have undergone the Alternate Test Procedure (ATP) outlined in the protocol of 20 June 1990. The methods are: automated steam distillation followed by Nesslerization, automated steam distillation with titrimetric detection and the automated Flow Injection Analysis (FIA) gas diffusion procedure. All TKN methods used aluminium block digestion for sample pre-treatment.

Final effluent samples obtained from ten sites representing the top five SIC classifications for each parameter were tested by each of the three methods. Data indicate that fast steam distillation gives results which are accurate and more precise than the accepted method. Further, the steam distillation process proved to be less time consuming and easier to operate even when compared to the ammonia electrode method.

The FIA gas diffusion procedure also showed improved results in addition to having an ammonia method detection limit (MDL) of 0.01 mg/l. The automated FIA method was straight forward and uncomplicated giving results in 70 seconds.

Data for each of the procedures were presented. The method approval process was discussed and outlined in relation to the submitted ammonia and TKN methodologies.

---

### Automated inorganic ion analyses for wastewater, sludge and stack gas incineration

*Aldo Conetta (Bran + Luebbe Analyzing Technologies, Inc., 103 Fairview Park Drive, Elmsford, NY 10523-1500)*

Both Federal and State EPA Agencies are mandating increased analytical testing for inorganic analytes. Knowing the level of contamination will often dictate the best remedial action required for cleanup. The end result is to significantly reduce contamination to the nation's waterways, soils and air for a healthier environment.

There is no sample matrix that is immune from these mandates, and, as each year passes, the number of organic and inorganic analytes being added to the hazardous waste list is increasing. For example, the US EPA Contact Laboratory Program has realized an approximately 500-fold increase in analytical testing in the past decade. In addition, there are approximately 3000 private laboratories involved in all aspects of environmental testing.

Automation for the environmental laboratory through instrumentation is the only alternative to meet analytical demands and still maintain a favorable cost/benefit ratio. Placements of such instrumentation have exceeded one billion dollars per year worldwide.

*Analytical instrumentation* – Analytical instrumentation can be categorized in many ways. However, with respect to environmental analyses a logical approach would be as follows:

(1) Laboratory analysers

(2) On-line process analyser

(3) Stack gas analysers

The one common thread for each of these analysers is automation. A review of analytical instrumentation currently available, coupled with specific applications was presented.

---

### Fast automated multi-element elemental analysis of sludges and effluents using a new sequential atomic absorption spectrometer

*William Batie and A. E. Bernhard (Analyte Corp., 910 Chevy Way, Medford, OR 97504)*

Over the past 10 years there has been a barrage of publications and papers extolling the desirability of methods alternative to Atomic Absorption Spectrometry (AAS) for the analysis of metals in sludge and effluents. Despite this, and the advantage of being essentially a single element technique, AAS continues to be widely used for this application because of its simplicity of operation and relative freedom from interferences compared to emission techniques.

Attempts have been made to automate AA resulting in the capabilities of analysing up to eight elements in a number of samples, one element at a time. This means that the analysis of the first sample of a group of samples is not complete until the last sample has been analysed. In addition, 8 elements are insufficient for this application, requiring operator intervention to change HCLs.

A new AA spectrometer, the ANALYTE 16, provides fully automatic analysis of up to 24 elements in a single pass on a sample at the rate of 10 elements per minute. This is achieved through use of a 16 hollow cathode lamp (HCL) turret which selects elements, one at a time, with a speed of 1 second. During the time the carousel is selecting a new HCL, the grating drives to the proper wavelength and the bandwidth is changed appropriately.

This novel instrument, and its use and capabilities for fast automated analysis of sludges and effluents, were discussed.

---

### Automated air analysis of volatile organic compounds on a new intermediate polar phase

*R. P. M. Dooper, D. Zwiep (Chrompack Inc., 1130 Route 202, Raritan, NJ 08869); and H. J. Th. Bloemen (National Institute of Public Health and Environmental Protection, P.O. Box 1, 3720 BA Bilthoven, The Netherlands)*

In recent years interest in air analysis has grown rapidly, especially for such volatile organics as ozone precursors. Automation of these analyses, usually done by capillary gas chromatography, is possible nowadays. To improve the reliability of these analyses, identification of the compounds of interest on two columns with different stationary phases often used. The most commonly used stationary phase is a 100% polymethyl silicone phase.

The best choice of a second column is one with a polarity between 5% phenyl/95% methyl silicone and an OV-17 type of phase.

This new phase has been developed. The phase is highly inert and chemically bonded. Also, the phase is especially useful in ECD applications.

---

### Automation of the USEPA's contract laboratory program requirements of the determination of 15 elements in a variety of environmental samples by inductively coupled Plasma Optical Emission Spectrometry (ICP-OES)

*Geoff Tyler, Flav Finotello and Tran Nham (Varian Optical Spectroscopy Instruments, 201 Hansen Court, Suite 108, Wood Dale, IL 60191)*

The requirement for strict quality control and quality assurance in the analysis of environmental samples is now well established and fully documented by the Environmental Protection Agency (EPA) Contract Laboratory Program (CLP). This need to ensure the validity and security of analytical data has increased the number of quality control test solutions required to be analysed.

This paper presented a Quality Control Protocol (QCP) software package designed for automation of sample analysis, QC testing, implementation of QC test failure actions and results reporting conforming to USEPA's

CLP requirements for ICP-OES. Comparison of detection and interference data with EPA recommendations shows that the package is applicable to a variety of environmental samples including wastewaters, potable waters, effluents and acid rain. Alternative elemental wavelengths providing higher analytical performance is recommended. The screening of sulphur in environmental samples ICP-OES was also proposed.

---

### Analytical performance of an automatic sample preparation system for atomic absorption spectrophotometry

*James A. Gaunt (Department of Civil and Construction Engineering, 123 Town Engineering Building, Iowa State University, Ames, IA 50011)*

Preparation of samples for analysis by atomic absorption spectrophotometry (AAS) frequently requires dilution, addition of matrix modifiers, and preparation of a range of calibration standards. Use of an automatic sample preparation system (prep station) can significantly decrease the time required for these activities.

The subject of this investigation was a PS-150 Prep Station used with an ISC-150 Intelligent Sample Changer and a TJA Smith-Hieftje 12 AA/AE Spectrophotometer (all components supplied by Thermo Jarrell Ash Corporation, 8 E. Forge Parkway, Franklin, MA 02038). Soon after the PS-150 Prep Station was placed in routine service, a quality assurance audit suggested that a study of the analytical performance of the prep station would be appropriate.

The gross volumetric accuracy of the prep station was tested by weighing distilled water delivered into tared sample tubes using the following protocol: 2·0 ml sample uptake (SU), 1·0 ml matrix modifier (MM), 5·0 ml final volume (FV). The average weight delivered was 4.9900 g (s = 0·0131, RSD = 0·263%, N = 12).

The sample dilution and matrix modifier and matrix modifier functions were tested by using known concentrations of $Cu^{++}$ as the 'sample' or the 'matrix modifier' in several different protocols so that the prepared solutions should have contained exactly 0·500 mg/l. Six replicates of each solution were prepared and subsequently analysed using flame AAS. Recoveries of $Cu^{++}$ were between 99% adn 102% for dilution ratios of 2×, 5×, 10× and 20×. A matrix modifier dilution ratio of 50x (SU=2.0, MM=0.1, FV = 5.0 ml resulted in a recovery of 108% (0·538 mg/l $Cu^{++}$).

The standard preparation function was tested by preparing six replicate sets of standards for both $Ca^{++}$ and $Cu^{++}$ using the prep station and analysing them by flame AAS calibrated with manually prepared standards. The results were as follows:

| Theo. mg/l Ca | 2·00 | 4·00 | 6·00 | 8·00 | 10·00 |
|---|---|---|---|---|---|
| Mean | 2·72 | 4·62 | 6·83 | 8·82 | 10·83 |
| s | 0·06 | 0·27 | 0·29 | 0·38 | 0·42 |
| Recovery (%) | 136 | 116 | 114 | 110 | 108 |
| Dilution ratio | 50× | 25× | 16·7× | 12·5× | 10× |

| Theo. mg/l Cu | 0·200 | 0·400 | 0·600 | 0·800 | 1·000 |
|---|---|---|---|---|---|
| Mean | 0·200 | 0·405 | 0·637 | 0·881 | |
| s | 0·001 | 0·004 | 0·003 | 0·009 | 0·003 |
| Recovery (%) | 100 | 101 | 106 | 110 | 103 |
| Dilution ratio | 50× | 25× | 16·7× | 12·5× | 10× |

The PS-150 Prep Station is a statisfactory instrument for preparing samples for analysis by AAS provided that the dilution ratio of sample or matrix modifier is not greater than 20× (i.e., uptake = 0·3 ml, final volume = 6·0 ml). However, it does not appear to be a reliable instrument for preparing calibration standards.

---

### Automatic continuous on-line analysis of haloforms in drinking water using process GC with electron capture detection

*Matthew Przybyciel (ES Industries, 8 S. Maple Avenue, Marlton, NJ 08053)*

Haloforms have been shown to be chlorination by-products of municipal water supplies. Possible haloform contamination have raised concerns over drinking water quality and has left to increased manual sampling for analysis. Unfortunately, manual sampling may not occur at frequent enough intervals to detect problems. In addition, manual sampling is extremely labour intensive and is prone to operator error.

The continuous automatic analysis of water using process GC with flame ionization detection has been commercially available for several years. The detection for many haloforms are in the 5–20 ppb range using this type of instrumentation. The levels are achieved using a continuous sparging technique along with the process GC. In order to achieve lower levels of detection for the haloforms using this type of instrumentation, electron capture detection will be required. Historically, electron capture detectors have not been stable or reliable enough for use in continuous automated instrumentation, however, the electron capture detector used in this study has been specially adopted for use in continuous operation.

In this paper the continuous detection of the haloforms at levels less than 1 ppb was shown. In addition, long term stability, reproducibility, cycle time and reliability of the instrumentation was discussed.

---

### Automated pre- and post-column reaction/detection systems in column liquid chromatography and capillary gas chromatography

*H. Lingeman,[1] U. A. Th. Brinkman[1] and E. A. Hogendoorn[2] ([1]Department of Analytical Chemistry, Free University, De Boelelaan 1083, 1081 HV Amsterdam, The Netherlands; [2]National Institute of Public Health and Environmental Protection, P.O. Box 1, 3720 BA Bilthoven, The Netherlands)*

In spite of the fact that column liquid chromatography (CLC) and capillary gas chromatography (CGC) are (becoming) mature analytical techniques, selectivity and sensitivity are still not always sufficient when solutes in relatively complex matrices (e.g. biological, environmental, food and fodder) should be determined. One of the possibilities to overcome this limitation is the use of a chemical reaction/detection (derivatization) procedure. These reactions can be performed in several modes: before, during and after the separation and in an on-line and off-line mode with CLC.

However, for routine analyses automation is one of the key parameters, which means that on-line methods are preferred over time-consuming and laborious off-line procedures.

Nowadays, a number of sample processors and continuous-flow systems are available allowing the automation of pre- as well as post-column reaction/detection and sample preparation techniques.

In this presentation the automated pre-column phase-transfer catalysed dansylation of phenolic steroids in plasma using a Model 231-401 sample processor, the automated on-line pre-column dialysis and concentration of benzodiazepine containing samples using a continuous-flow system (ASTED), the combination of an on-line post-column derivatization and on-line pre-column dialysis procedure of the determination of sulfonamides in food products, and a clean-up and detection system for the determination of polar pesticides in aqueous samples using automated (ASPEC) ion-pair derivatization with pentafluorbenzylbromide and capillary gas chromatography with electrochemical detection was discussed.

Analytical variables (e.g. reproducibility, repeatability, throughput, detection limits) of the different methods were compared.

---

### On-line analysis of moving solids by transient infrared spectroscopy

*Roger Jones and John McClelland (Center for Advanced Technology Development and Ames Laboratory-USDOE, Iowa State University, Ames, IA 50011)*

Industry requires methods to quantitatively and non-destructively analyse solid materials in real time as they move along a production line. Transient Infrared Spectroscopy (TIRS) can provide such analyses. In TIRS a thin layer of the sample material is transiently heated or cooled, usually by a gas jet, as it passes through the field of view of an infrared spectrometer. This surface layer is spectroscopically different from the bulk of the material, and it may be analysed separately from the bulk. If the layer is heated, it acts as a thin emission source with much less self-absorption than is characteristic of thick emission sources. If the layer is cooled, it acts as a thin absorption sample through which blackbody emission from the bulk of the sample passes on its way to the spectrometer. In both cases, the layer is thin enough that saturation is reduced sufficiently for quantitative analysis.

A protype industrial-site instrument is presently being developed for on-site test and demonstration. It is based on a Bomem MB-100 FTIR spectrometer fitted with special sampling optics. A commercial hot-air tool with a modified nozzle is used as the surface-heating source. The properties and performance of the prototype were discussed.

Laboratory results demonstrating some of the special properties of TIRS were also covered. TIRS can be done with only moderately hot (below 100 °C) or cold (above −50 °C) gas jets, as long as the heating or cooling is rapid enough to create a suitably thin transient layer. The performance at various jet temperatures will be presented. As the figure below demonstrates, the morphology of non-isotropic materials can be determined by using polarization-sensitive detection. Depth profiling can be performed by controlling the thickness of the thermally altered layer at the time of observation. The freedom of TIRS from interference effects (fringes) when the sample material is thin and has parallel sides was also shown.

---

### Determination of vitamin C content in pharaceuticals via flow-injection chemiluminescence detection

*John Vicars and Chu-Ngi Ho (Department of Chemistry, Box 23, 350A, East Tennessee State University, Johnson City, TN 37614)*

Because of the nutritive and other perceived health value of vitamin C, commercial sources of the vitamin are numerous and availability abundant. Because of its importance, many methods have been developed for the determination of vitamin C in all sorts of samples ranging from pharmaceuticals, agricultural and food products to biological fluids. The methods include classical titrimetry, ultra-violet and visible absorption, luminescence spectrometry and high performance liquid chromatographic separation.

The authors have developed and implemented a simple and rapid flow injection system coupled with chemiluminescence (CL) detection to analyse pharmaceutical samples for the amount of vitamin C present. The CL system utilized is that of peroxyoxalate employing bis(trichlorophenyl)peroxyoxalate (TCPO). The vitamin C in different samples is reacted with a given fixed amount of hydrogen peroxide in the presence of Cu(II) as catalyst. Imidazole which catalyses the CL reaction is added to the solution. The excess hydrogen peroxide can then react with TCPO to produce the CL using perylene as fluorophore. The TCPO and perylene are premixed and pumped into a flow system. The vitamin C and excess hydrogen peroxide incubation mixture is injected through a 20 μl Rheodyne injector loop into the flow stream. The reaction to produce CL takes place in the flow and the signal is detected in a homemade flow cell placed in front of a photomultiplier tube. The TCPO and perylene are made in methylacetate while the reaction mixture is entirely aqueous. There does not seem to be any mixing problem at all. In this presentation, the procedure, optimization of the solvents used, flow rate, and concentration of different reagents, data and the results obtained were discussed.

---

### Extending the capabilities of flame atomic absorption spectrometry with on-line sample treatment

*S. R. Bysouth, J. F. Tyson, E. Debrah, T. J. Gluodenis and R. M. Larue (Department of Chemistry, University of Massachusetts, Amherst, MA 01003)*

Many of the limitations to the performance of flame atomic absorption spectrometry (FAAS) can be accounted for by (*a*) shortcomings in the nebulization and atomization processes and (*b*) interference effects due to matrix components. For a number of years, research in the authors' group has been directed towards overcoming these limitations by the use of continuous-flow and flow injection procedures of sample handling. Three uses have been made of these techniques: (i) separation of the analyte from its matrix, (ii) preconcentration of the analyte and (iii) generation of volatile analyte species. Examples of recent work in each of these areas were presented to illustrate the potential of on-line, sample treatment procedures. The determination of trace elements in silver as silver chloride with on-line filtration provides an example of the first procedure where the matrix is removed. The alternative version of this system, where the analyte is precipitated, was demonstrated for the on-line preconcentration of copper as the hydroxide as an example of the second procedure. On-line generation of a volatile metal complex where the complex is subsequently extracted into supercritical or liquid carbon dioxide before direct introduction to the atomizer involves all three procedures. Vaporization of the carbon dioxide carrier stream can be achieved using a simple heated restrictor. This device has been shown to produce calibration curves with sensitivities comparable to that obtained with continuous nebulization, when only 20 μl of a solution of the complex in a solvent is injected. Results of the investigation of the separation of the complex from the aqueous phase by solid phase extraction or precipitation followed by re-dissolution in carbon dioxide and direct liquid–liquid extraction were presented.

The extension of such procedures for use in conjunction with electrothermal atomization AAS were also discussed.

---

### Method studies in ambient air analyses

*Dick Siscanaw (US EPA, Region 1 Laboratory, 60 Westview St., Lexington, MA 02173); Hui Wang and Delon Maas (Roy F. Weston, Inc., Landmark One, 1 Van de Graaft Dr., Burlington, MA 01803)*

In environmental laboratories, most of the work has historically been water, soil, and drum samples. Now measurements for organic air toxics are increasingly being requested. The authors' load has more than doubled in the past three years. For air, the laboratory faces a variety of situations which have different data quality objectives and require different methodologies. For example, an emergency is treated differently from an ambient air study at a superfund site.

Five years ago, most of the authors' air analyses were for emergencies and an occasional site investigation. The portable gas chromatograph was used approximately 90% of the time. The remaining techniques were the Tedlar bags, color detector tubes, and NIOSH methods. These techniques are still used today. The practical quantitation levels (PQLs) ranged from 10 ppb for the volatile aromatics to ppm. This was relatively high for some ambient site investigations.

In 1986, Region 1 started measuring the volatile organics using the Spherocarb and Tenax solid adsorbents, EPA methods T01 and T02. A measured volume of air is drawn through the trap, typically 5–20 l, which is later thermally desorbed into the gas chromatograph/mass spectrometer (GC/MS). Our PQLs dropped by 50 ppt. Last year, the laboratory expanded its air program to include the SUMMA passivated canister, EPA method T014. In this method, 500 ml is drawn into the GC/MS for analysis. QLs for the canister is approximately 2 ppb.

This presentation focused on experiences with these methods and the modifications made. The subjects included are field analyses and screening, various samplers (sector sampler), quality control (replicates, back-up, and distributive samples), cleaning apparatus, and the preparation of our standards.

---

### Automatic perimeter monitoring of airborne organic compounds at a superfund remediation site utilizing a gas chromatograph equipped with a multipoint sampling system

*Amos Linenberg (Sentex Sensing Technology Inc., 553 Broad Avenue, Ridgefield, NJ 07657)*

Since the US National Priority list for the clean-up of the nation's most highly contaminated sites has been created, EPA has established funding priorities and made the advancement of sites to the construction phase a main goal of the EPA Superfund Program.

Many of thse sites are located near residential areas, therefore, it is necessary to determine the identity and quantity of the pollutants that escape the confines of the remediation site. Usually, this is accomplished by obtaining samples at various locations along the perimeter of the site and transporting the samples to a central laboratory for analysis. This procedure is time consuming, and often leads to inaccurate results due to loss of sample during transportation.

A monitoring system based on an automatic multipoint has chromatograph has been installed at a landfill, which is ranked among the top Superfund sites on the National Priority list.

This system automatically draws ambient air samples from 16 different sampling points along the perimeter, injects each sample into the gas chromatograph, performs the analysis, and stores the data on a computer disk. The frequency and order in which each site is sampled is operator controlled.

A specially developed software program enables the operation of the system, as well as obtaining parameters and retrieval of data from a remote location through the use of a second computer and a modem.

The description of the operation of the system, its set-up, configuration and performance was discussed.

---

### Alternate sampling strategies for ICP-MS

*R. C. Hutton, D. Gregson (VG Elemental Limited, Ion Path, Road Three, Winsford, Cheshire CW7 3BX, UK); W. Vogel, J. D. Steiner, A. Eastgate (ARL, En Vallaire, CH-1024 Ecublens, Switzerland)*

The analytical potentials of ICP-MS have been expanded by the capability of utilizing a variety of sample introduction techniques. To date in commercial terms, both laser ablation and electrothermal vaporization are available.

There are, however, still many other sampling techniques which can further enhance analysis by ICP-MS. Save for the use of ultrasonics, the solutions nebulizer has not undergone any significant technical advancement, yet this area is a key to analytical performance.

A nebulization system which effectively removes 99% of all solvent and enhances ICP-MS sensitivity by up to 40× has been developed. The applications for this device for the analysis of high purity reagents will be discussed.

Analysis of metal samples can be carried out by LA-ICP-MS. However, there are application areas where laser technology may be too sophisticated and expensive. Preliminary results from the coupling of a conductive solids nebulizer customized for ICP-MS was described. In particular, the performance in a metals process control environment was evaluated.

---

### Automated analysis of industrial waste effluents using photo-oxidation with ultraviolet radiation

*R. B. Roy, John Swanson and Shirley Arment (ALPKEM Corporation, 14625 S.E. 82nd Drive, Clackamas, OR 97015)*

Industrial waste effluents are analysed to (i) determine the compliance of wastewaters, (ii) estimate the toxic effects of waste discharges, and (iii) evaluate the pretreatment requirements for reuse of water.

Most frequently, organic carbon, chemical oxygen demand, nitrogen, phosphorous, cyanide and chromium are determined in a wide variety of industrial waste effluents. For the measurement of these parameters, samples are oxidized using either wet chemical oxidations or ultraviolet-promoted photo-oxidation procedures. The resulting breakdown products, such $CO_2$ and $NH_3$, are determined to establish organic carbon and nitrogen contents, respectively, present in the samples.

The ultraviolet-promoted oxidations are simple and avoid problems which are associated with wet chemical oxidations and combustion procedures. Photo-oxidation procedures are also used to clean up colored samples for the measurement of metal ions.

In this presentation, the application of UV-promoted photo-oxidation procedures used for the measurement of organic and inorganic materials present in a wide variety of waste effluents were highlighted. In addition, selection of experimental conditions, interferences encountered and their elimination were presented.

---

### Automated analysis of reducing sugars in fermentation broth

*R. B. Roy, Shirley Arment and John Swanson (ALPKEM Corporation, 14625 S.E. 82nd Drive, Clackamas, OR 97015)*

Various sugars, molasses and corn syrups are used as carbon source for the growth of microorganisms in a wide variety of industrial fermentation processes. Measurement of reducing sugars ensures the microbial growth and product formation during the operations of industrial fermenters.

The fermentation broth consists of insoluble, gelatinous biomass, the nutrient fluid, and the soluble metabolites resulting from the fermentation operation. Due to the presence of polysaccharide materials in biomass, the fermentation broths are, usually, viscous and sticky. Samples are diluted as filtered prior to analysis.

Usually, manual colorimetric, liquid and gas chromatographic procedures are used for the determination of reducing sugars from fermentation broths. For rapid and reliable analysis of reducing sugars, an automated method using an ALPKEM RFA system was developed. Samples are dialysed and the dialysates are reacted with alkaline potassium ferricyanide. The decrease in absorbance, which is proportional to the amount of reducing sugars, is measured at 420 nm.

The results obtained using the RFA system compare well with standard manual methods. The method is sensitive and can be used to determine reducing sugar content of a wide variety of fermentation broths. In addition, the procedure developed has the potential for on-line monitoring of reducing sugar contents present in fermenters and/or bioreactors. Thus, ALPKEM RFA system could be used as a part of closed loop system in which the output from the analyser could be used to automatically control reducing sugar variations in fermenters. Technical details needed in the transportation of samples to the RFA system were also discussed.

---

### Total cyanide analysis by manual standard methods and automated Kelada method, comparative study and data evaluation

*Nabih P. Kelada (Head of Methodology & Toxic Substances, Metropolitan Water Reclamation District of Greater Chicago); R & D Egan (550 S. Meacham Rd., Schaumburg, IL 60193); and Cecil Lue-Hing (Director of R & D Dept., Metropolitan Water Reclamation Distriuct of Greater Chicago)*

An automated method for total cyanide has been developed (20 samples/h). In addition to the Bran Lube (Technicon Industrial Systems) AutoAnalyzer II modules for segmented flow, the system utilized two new automated units, for ultraviolet (UV) irradiation and for continuous thin film distillation. The UV irradiation is performed in alkaline medium and Pyrex sample coil, thus the strong cyanide complexes (Fe and Co) are effectively broken down and measured colorimetrically, but not thiocyanate.

The automated Kelada Method is making good progress for acceptance by the American Society for Testing and Materials (ASTM). The round robin study for performance evaluation has been completed successfully and gave about 5% coefficient of variation, and 90–100% spike recovery for most sample matrixes. The method, as found by the participants, is applicable to water, wastewater (effluent, raw sewage and sludge) and industrial discharges. Eventually this method will replace the present ASTM D-4374 for automated total cyanide.

The present USEPA approved Method 335-2 (same as Standard Method 412-B and ASTM D-2036-A) is time consuming ($\geqslant 1\frac{1}{2}$ h/sample), has wide variability and frequently subjected to thiocyanate interference. An in depth study was conducted to compare the manual approved method with this auomated method. Samples analysed were collected from sewage treatment plants (for National Discharge Elimination System, NPDES), and other cases as stated by regulations 40 CFR (aluminum forming, coil coating, electroplating, organic and inorganic chemicals, iron and steel, metal manufacturing and powders, and petroleum industries). Spikes included simple and strong complex cyanides (100–5,000 μg/l). Results obtained by both methods show that they are comparable. However, other than being free from thiocyanate interference, the automated Kelada Method consistently gave far better detection limit (0.5 μg/l), precision, accuracy and spike recovery. Representative data were presented.

---

### Water quality control via on-line total organic carbon determination

*Steve H. Vien, Connie M. Courtney, Walt W. Henslee, and John M. Leach (Dow Chemical USA, Analytical & Engineering Sciences, Freeport, TX 77541)*

The analysis of total organic carbon (TOC) is important to assess the water quality in a water treatment facility and in chemical processes which do not tolerate high organic content. In addition, TOC analysis of the incoming stream in a water treatment plant helps protect microorganisms from organic shock. A composite sampling technique followed by laboratory analysis of the daily composite sample is commonly used. This method is generally too slow to respond to plant upsets.

An on-line TOC analyser has been recently evaluated in the authors' laboratory and on a process stream. This instrument makes use of the low temperature catalytic air oxidation process to oxidize organics. The instrument features a unique variable sample volume injection and multi-ranging system which eliminates the need for sample dilution. Sampling cycles for as short as 3 minutes can be achieved. The principle of operation, analytical performance of the instrument, and the results of field applications were presented.

---

### An automatic method to measure agglomerated and dispersed particle size and size distribution

*Jason Ruan (Reichhold Chemicals, Inc. P.O. Drawer 'K', Dover, DE 19903)*

Dozens of methods for measuring particle size distribution (PSD) have been developed in the past decades. Among them, microscopic techniques are still the most reliable and are the industry standard. The drawback of these techniques, however, is that they are slow and tedious. Though much progress has been made since the advent of the image analyser, they have not been successfully applied to resolve agglomerated particles. Even the very sophisticated systems with the deagglomeration feature can only analyse very narrow distribution and barely touching particles.

A method has been developed for measuring agglomerated, as well as dispersed, PSD from electron micrographs. The chord lengths of horizontal lines scanned through the micrographs are measured. The chord distribution is converted to PSD as if all particles were deagglomerated. A mathematical procedure has been derived for converting chord distribution, g(l), to PSD, F(x). Non-linear Gaussian regression curve fitting was also applied.

The technique has been verified by computer simulation and implemented on a variety of latex samples. They all show that this technique gives excellent representation of the actual particle size distribution. The whole process is completely automated and takes less than a minute instead of several hours. This method is specially designed to resolve agglomerated particles, but it is also applicable to micrographs of fracture surface for the core-shell structure and/or solid foam analysis.

---

### Automated sample handling and quantitation in lubricant analysis

*Jay R. Powell and David A. C. Compton (Bio-Rad, Digilab Division, 237 Putnam Ave., Cambridge, MA 02139)*

The analysis of lubrication oils for wear metals by atomic spectroscopy and oil additive depletion, breakdown and contamination by infrared spectroscopy has been a long established technique in this field. The analysis of wear metals by atomic spectroscopy lends itself to automation easily because of two major factors. First, the quantitative analysis is relatively simple, because atomic absorption of a given metal will most often follow a linear Beer's law fit, or an easily corrected deviation. Second, the relatively viscous oil can be easily diluted with a solvent such as kerosene in order to permit aspiration into the flame or plasma, with the dilution ratio determined by the use of an internal standard. By contrast, creating an automated system for the analysis of lubricants by infrared spectroscopy has been much more difficult. Quantitative analysis of these fluids is complicated, due to the complex nature of these fluids in terms of both the number of components and interactions between all the components. Recent advances in multivariate quantitative techniques has some promise in overcoming these difficulties. In addition, sample handling is complicated by the fact that no dilution solvent exists that is soluble in the oil and is infrared inactive. This requires that the sample be transported neat to the sample cell, with a non-interfering solvent used to clean the cell and transport system between samples. Here, the design of an automated infrared lubricant analysis system was discussed, dealing with both a comparison of classical quantitative techniques to multivariate techniques, and how component architecture can affect sample handling and transport in a commercial system.

---

### Designing optimized industrial process analysers for closed loop control

*Hanno Kellner, Gerd Popp and Uwe Grummisch (Bran + Luebbe GmbH, Postfach 1360, Werkstrasse 4, D 2000, Norderstedt, Germany)*

Until now the day-to-day application of near infra-red has been primarily focused on laboratory and 'at-line' analysis. However, the benefits of this technology are most dramatically realized when the analysis takes place directly in the process line.

Process conditions vary from explosion proof environments in the chemical industry to clean-in-place environments in the food and pharmaceutical industries. Therefore, installation of near infra-red analysers requires optimization of both the analyser design and the sampling interface. Different approaches will be discussed including filter based systems and fiber optic based scanning systems.

Full closed loop process control is achievable when this analysing approach is combined with precision dosing systems, weighing electronics and process control units. Real world examples from various types of industry were included in the paper.

---

### On-line multiparameter process analysis utilizing flow injection procedures

*Paul Karges and Karl G. Schick (Eppendorf North America, Inc., 545 Science Drive, Madison, WI 53711)*

Process analysers must be rugged and dependable. Versatility is usually not as important. Sometimes, however, novel chemistries can be combined with appropriate hardware to satisfy the requirements for ruggedness and yet are simple enough so that two parameters can be measured dependably. Two multi-parameter Flow Injection Analysis (FIA) methods were described, both of which use a single wavelength colorimetric detector and a single manifold.

The first application determines acidity (up to 25 ppm HCl) and basicity (to 40 ppm KOH) of a complex organic matrix, 70% of which is toluene. Bromocresol purple indicator dissolved in 1-butanol is used as the carrier. A colorimeter operating at 600 nm is used as the detector. When the carrier is properly adjusted, an increase in the carrier absorbance is proportional to the concentration of potassium hydroxide while a decrease in absorbance is proportional to the concentration of hydrochloric acid.

The second application consists of the measurement of the concentration of zinc sulphate (0·1–6%) and sulphuric acid (2–9%) in a synthetic fibers plant. For this application the normal FIA mixing coil is replaced with an exponential dilution chamber followed by a colorimeter operating at 540 nm. A three-way solenoid valve alternatively switches between the zinc carrier, which consists of xylenol orange dissolved in a mixed buffer solution, and the acid carrier, which consists of the same buffer containing a methyl orange indicator.

An explanation of the exponential dilution chamber, along with a description of how the technique of electronic dilution was used to extend the range of the zinc sulphate analysis, was given.

---

### Automated real-time response surface mapping for continuous flow systems

*Peter D. Wentzell and Nils G. Sundin (Trace Analysis Research Centre, Department of Chemistry, Dalhousie University, Halifax, Nova Scotia, Canada B3H 4J3)*

The development of continuous flow methods of analysis often requires the optimization of numerous experimental parameters. Because of the complex interactions among these variables, empirical optimization strategies, such as the simplex method, are usually preferred when the primary objective is to obtain the optimal operating conditions. If the objective is to obtain a better understanding of variable interactions, however, systematic response surface mapping (i.e. response vs. experimental variables) with an appropriate experimental design is more useful. One way to perform both of these tasks efficiently and reliably is through the use of a computer-controlled continuous flow apparatus. In this talk, the implementation of a highly automated continuous flow system constructed in our laboratory was discussed.

One of the drawbacks to existing response surface methods is that they are normally carried out off-line, i.e. after all experiments are complete and the apparatus has been shut down. While this permits fairly comprehensive data analysis, it eliminates any kind of immediate experimental feedback. The authors have developed software which allows three-dimensional response surface maps to be viewed while the experiments are in progress. This provides some distinct advantages, particularly for investigating noisy, suspicious, or unusual regions of the surface. Also, since the time lag between experiments is small, conditions are more reproducible. The details of this approach were illustrated through its application to a hydride generation/atomic absorption system for the determination of arsenic.

Response surface display methods employ several strategies, all of which possess certain advantages when applied to experimental systems. The most basic method is the generation of 'stick plots' in which the data are displayed as vertical lines in a 2-dimensional projection of a 3-dimensional space. This has the advantage of providing a true representation of the measurements, but gives a poor perspective on the overall shape of the surface and is not as visually pleasing as the more common 'wire frame' representations. The generation of wire frame surfaces or topographical maps requires that the data be modelled however, and this always runs the risk of imposing artificial structure on the data. Polynomial modeling and triangulation with simple or complex interpolation are the most common approaches currently used. Because the ideal modeling method depends on the nature of the chemical data set, the automated system developed in our lab makes a variety of techniques available. This permits more thorough examination of variable interactions while the experiment is in progress. Methods for improving the efficiency of the mapping procedure through alternatives in experimental design have also been considered.

---

### On-line process analyser for chemically bound nitrogen and sulfur compounds

*James S. Wreyford (Antek Instruments, Inc., 300 Bammel Westfield Road, Houston, TX 77090)*

Bench top analyses of nitrogen compounds by pyrochemiluminescent nitrogen specific detector and sulfur compounds by pyrofluorescent sulfur system have been successfully utilized in various academic and industrial laboratories for several years. The need for on-line nitrogen and/or sulfur analyses in the industrial food and chemical processing plants and refineries prompted development of an on-line process system using continuous flow and/or discreet sampling techniques.

The analytical techniques, sensitivity, as well as linearity and repeatability of response were discussed for water, liquid hydrocarbons, and gas applications.

---

### A new method for chromium determination in chrome-phosphate treatment on aluminum surface

*Richard F. Puchyr and Ravi Raja (American National Can Company, 433 N. Northwest Highway, Barrington, IL 60010)*

The passivation of the surface of aluminum coils used for production of food cans is crucial for good adhesion between the aluminum and the organic coating applied to the metal surface. A chrome-phosphate chemical treatment is applied to aluminum coils for this purpose. Chromium content seems to be the most important factor that determines the adhesion.

There are methods using X-ray fluorescence spectroscopy to determine the amount of treatment on aluminum coils, but all require known aluminum plate standards. There has not been an established method to determine the amount of chromium in the treatment on an aluminum surface. Since different aluminum suppliers use different formulations, the same weight of treatment from two suppliers may not have the same chromium content. Therefore, it is important that there is a method to determine the chromium content itself when no known plate standards are available. Once the chromium content is known the chemical treatment levels can readily be determined by any supplier.

The authors have developed a method for this purpose. This method uses a combination of atomic absorption and direct reading emission spectroscopic techniques. The method consists of removing the treatment using hydrochloric acid and determining the chromium content in the acid solution by an atomic absorption procedure. A small amount of chromium in the aluminum that is usually removed with the chromium treatment contributes to the chromium content determined by AA. A correction for this is made by determining the alloy chemistry of the coil by a Direct Reader emission spectrometer and compensating for the chromium contribution from the alloy that dissolved into the acid solution.

As an offshoot of this method, the authors have also derived a fast indirect method for determining the relative treatment levels on the aluminum coil by using a Direct Reader emission spectrometer, using known standards.

---

### The automation of an atomic absorption spectometer for on-stream analysis of total iron in zinc sulphate solutions

*Michael Knowles, Fred Delles (Varian Optical Sectroscopy Instruments, 201 Hansen Court, Suite 108, Wood Dale, IL 60191); D. L. Hoobin (Vrian Pty. Ltd., 11–13 Palmer Court, Mount Waverley, Victoria 3071, Australia); B. R. Champion and M. J. Howell (Pasminco Metals-EZ, GPO Box 377D, Hobart, Tasmania 7001, Australia)*

This paper described the design of an automated on-stream analysis system for the determination of total iron in zinc sulphate solutions. The Pasminco Metals–EZ Neutral Leaching Plant removes iron from zinc plant electrolyte by leaching impure zinc oxide with acidic spent electrolyte. An iron dosing solution is supplied to the plant to maintain the Fe(III) concentration at the desired level.

A fully automated system was described which is programmed to periodically sample plant solution and prepare the sample for analysis by an autotitrator for Fe(II) and an atomic absorption spectrometer (AAS) for total iron. The Fe(III) level is calculated as the difference and is used to control the flow of iron dosing solution. The AAS has provision for user written software for instrument control via a GP10 communications utility, which handles up to 12 digital outputs and 14 digital inputs, 2 RS232C ports and an HP-GPIB port. An Opto 22 solid state Input/Output (I/O) interface was assembled to activate the flame and the stream selector system during each analysis.

Other I/Os synchronize and advise analyser status to the plant-wide Taylor MOD 300 Distributed Control System (DCS). Customized software had been prepared for the AAS system to control the analysis and to allow the programming of additional VDU displays and computations and for the computed results to be sent to the MOD 300 DCS via an RS232C data link.

Aspects of the system design and implementation were discussed, including the software design approach, the details of the analysis and aspects of interfacing and safety.

---

### Intelligent automated laboratory systems

*H. M. Kingston, F. A. Settle,* M. A. Pleva,** P. J. Walter, G. W. Kramer and L. B. Jassie (Consortium on Automated Analytical Laboratory Systems (CAALS), National Institute of Standards and Technology, Center for Analytical Chemistry, Inorganic Analytical Research Division, Gaithersburg, MD 20899; *Department of Chemistry, Virginia Military Institute, Lexington, VA 24450; **Department of Chemistry, Washington and Lee University, Lexington, VA 24450)*

Industrial and government operations both depend heavily upon the analytical laboratory for chemical measurements. These measurements provide critical feedback in research, acceptance testing, environmental quality, and quality assurance. The field of chemical analysis has become instrument based and is now capable of being integrated into an automated laboratory environment. Integrating instrumentation into analytical systems that automate complete analyses is difficult due to incompatibilities in hardware, software, and sample transport. Several key issues have been identified that will have a positive effect on the development of more efficient instrumentation in the automated laboratory. These issues include instrument modularity, intelligence, communication, and integration and must be addressed to provide the highest level of sophistication for the laboratory.

The National Institute of Standards and Technology (NIST) has joined with US industry to form the Consortium on Automated Analytical Laboratory Systems (CAALS). The Consortium seeks to accelerate the development of automated analysis methods and systems, to improve efficiency and data quality, and to promote transferability of analytical methods.

The automated systems developed will integrate chemical analysis expertise (intelligence), provide recommended methods for utilizing modular analytical instrumentation, and incorporate quality assurance procedures into the automated methods.

The first component of the system, an automated microwave decomposition station, has been developed. It can be controlled by a prototype expert system that is being developed to assist the analyst in establishing procedures for microwave dissolution. The entire system will consist of three modula designed components. These components represent fundamental steps in inorganic analysis, specifically analysis release, separation and detection. The specific modules that will be combined to perform these functions are microwave decomposition, chelation chromatography, and ICP-OES and/or MS. The system was discussed emphasizing intelligent control.

---

### Intelligent, automated laboratory systems

*Frank A. Settle, Jr (Department of Chemistry, Virginia Military Institute, Lexington, VA 24450)*

Although many laboratories have demonstrated that the automation of one or two steps of an analytical method can improve both the quantity and quality of results, few methods have been totally automated. Critical problem areas in implementing automated methods are method development and method validation. The resources required for automation are often lacking in laboratories where automation is needed most. Once an automated method has been validated it can be transferred electronically among laboratories with identical automation components.

An intelligent laboratory system to expedite the design, optimization, validation and transfer of automated analytical methods was described. This system integrates existing software for experimental design, statistical analysis, expert system development and laboratory automation with the components required for sample preparation and analyte measurement. Although most of the components are commercially available, it has been necessary to develop programs to facilitate communication among components.

The hardware and software components should be compatible and interchangeable to facilitate efficient configuration, optimization and transfer of automated methods. The preliminary results from colorimetric determinations of orthophosphates in water samples were presented.

This work was funded in part by NSF Grant #CHE-8805930, Zymark Corporation and a grant from the VMI Foundation.

---

### Flow injection analysis of glutamate with a glutamate oxidase reactor and acridinium ester chemiluminescence detection

*Maureen Stuever Kaltenbach and Mark A. Arnold (Department of Chemistry, University of Iowa, Iowa City, IA 52242)*

Glutamate is believed to be the primary excitatory neurotransmitter in the mammalian central nervous system. Despite its importance, the physiology of glutamate in neurotransmission is not well understood. A typical neurochemical study for glutamate involves perfusing a slice of nerve tissue with fluids containing agents known to alter natural glutamate uptake and release mechanisms. Aliquots of fluid are removed from the tissue and analysed by HPLC for changes in glutamate levels. These analyses typically require 30 minutes per sample, making a single experiment last several hours. The purpose of our work is to develop a fast, selective glutamate analysis method which is sensitive in the 1 to 10 micromolar concentration range in order to facilitate the study of glutamate as an excitatory neurotransmitter.

The authors' method relies on the principles of flow injection analysis for high sample throughput and precise measurements. The use of a glutamate oxidase enzyme reactor coupled with acridinium ester chemiluminescence detection provides excellent selectivity and sensitivity. The chemistry of our method is outlined below where PMAC (phenyl 10-methylacridinium-9-carboxylate methosulfate) represents the acridinium ester used in this project. The method uses a merging zones approach to conserve the acridinium ester. Two buffer carrier streams are pumped continuously and the ester is injected into one stream and the sample into the other under precise time control. The sample passes through a glutamate oxidase reactor where glutamate is converted to peroxide. The peroxide and ester meet at a mixing 'T' and hydroxide merges with the mixture just before the detection chamber. The resulting light is detected by a photomultiplier tube.

$$\text{Glutamate} + O_2 + H_2O \xrightarrow{\text{glutamate oxidase}} \alpha\text{-ketoglutarate} + NH_3 + H_2O$$

$$H_2O_2 + \text{PMAC} + \text{NaOH} \longrightarrow \text{n-methylacridone} + CO_2 + h\gamma$$

*Figure 1. Outline of reaction scheme.*

The first phase of the project involves an investigation of second reaction above. The peroxide/acridinium ester chemiluminescence detection method is incorporated into a flow injection analysis configuration. In the method development, the parameters of interest include temperature, operational pH, storage pH, carrier flow rate, ester concentration, hydroxide concentration, and selectivity over amino acids and ascorbic acid. Results show that peroxide concentrations ranging from 50 nanomolar to 0.1 millimolar can be analysed at a rate of about 240 samples per hour.

The second phase involves automation of the peroxide FIA, development of an effective glutamate oxidase reactor, and characterization of the coupled glutamate oxidase/acridinium ester FIA system. Four nylon tube immobilization procedures were compared to an immobilization onto porous glass beads. The porous glass beads are packed into a 3 cm column and produce superior glutamate conversion efficiency compared to the nylon tube reactors. The parameters of interest for the glutamate FIA are similar to those in the first phase of the project. To demonstrate the analytical utility of the method, the authors analysed samples collected during a pharmacological toxicity assay and compared these results to those obtained with the standard HPLC technique.

---

### An automated system for the analysis of trace metals by anodic or potentiometric stripping

*Sandra K. Wheeler, Thomas H. Ridgway and William R. Heineman (Department of Chemistry, Mail Location 172, University of Cincinnati, Cincinnati, OH 45221-0172)*

Anodic stripping voltammetry (ASV) has been a widely used analytical technique for the determination of trace metals for many years. A somewhat less common technique, potentiometric stripping voltammetry (PSV), may be more analytically appealing than ASV in many instances. Here, the reduced preconcentrated metals are reoxidized chemically instead of voltammetrically and are therefore quantitated in the order of their inherent redox potentials, where the time the electrode remains at each potential is proportional to the concentration of each species present. Since both current-potential and time-potential relationships can be relatively easily digitally monitored, automation of a computer driven stripping system is relatively straightforward and inexpensive. An IBM-compatible PC-based system and flow cell have been designed for anodic or potentiometric stripping analysis, capable of the sensitive and selective determination of heavy metals at low concentrations. The hardware consists of a potentiostat, A/D converter, D/A

converter, one 6-way and two 2-way electronically actuated mixing valves and two 3-way electronically actuated isolation valves, with associated digitally controlled valve drivers that have been interfaced to an XT class PC. Electronic waves permit up to ten separate solutions to be used, including a Hg plating solution, chemical oxidants for PSV (e.g. $Hg^{2+}$), a Hg film removal solution, electrode washes, buffers, and sample solutions that are all sequenced under computer control. The flow cell for this system consists of a glassy carbon working electrode (area = 0·264 $cm^2$) housed within a polyether-etherketone (PEEK) body to resist chemical degradation. This cell was specially designed for easy access and cleaning and requires minimal volumes (7 μl), which is of obvious benefit for the analysis of small volume samples. Evaluation of anodic and potentiometric stripping performance and the advantages of each operational mode was presented.

---

### An automated ICP method for the on-line analysis of aluminum alloys

*John L. Genna, Michael L. Ruschak, William D. McAninch, and William C. Craig (Aluminum Company of America, Alcoa Laboratories, Alcoa Center, PA 15069)*

The analysis of aluminum for production control is tradionally done using solid samples taken directly from a furnace or trough. These samples are sent to a remote laboratory for analysis by high or low voltage spark excitation optical emission spectroscopy. This type of analysis is well suited for low casting rate, batch processes but cannot deliver the timeliness nor volume of samples required for the control of the newly developing continuous processes or the monitoring of compositional uniformity required in today's very high volume batch casting procedures.

An on-line system has been developed that samples molten aluminum directly from a casting trough using a rotating copper wheel with sawtooth-like serrations around its circumference. Metal freezes on the outer edge of the wheel in the form of small flakes which are removed from the wheel using a high velocity gas stream and transported pneumatically to a remote, automated sample preparation system. Under computer control the sample is dissolved in an $HCl/H_2NO_3$ mixture and transported directly to a simultaneous ICP system which is also under direct computer control. Once initialized, the system is completely automated and requires no operator intervention.

Sampling frequency has been increased approximately 20 to 30 times over traditional manual sampling and analysis. A sample and analysis can be obtained every two minutes without sacrificing analytical precision or accuracy when compared to conventional analytical techniques. Both major and minor elements can be monitored in a near real time fashion.

---

### The use of statistical quality control techniques to monitor data that is censored and/or not normally distributed

*John H. Sheesley, Kenneth J. Anselmo and Jack V. Martinez (Air Products and Chemicals Inc., Allentown, PA 18195)*

The astounding successes of the Japanese quality effort in the 1970s caused a reemphasis on quality improvement in the US during the 1980s to reverse, or at least cease the widening of, the quality gap. The focus was placed on quality system deployment, system audit, quality motivation, the quality improvement process and, of course, statistical control.

Statistical control is the quality tool kit used for monitoring, troubleshooting and improving processes and products. It is a vital element of the quality system. It is a central point of the Deming and Juran quality philosophies.

One, if not the, prime element of statistical control is the control chart, developed in the 1920s by Shewhart. It is simple to use but profoundly powerful. It can be applied to attributes as well as measurements data; however, for measurements data the traditional procedures require that the data not be truncated or censored. When measurements data are censored; such as is the case with readings below the detection limit of the measuring equipment, or stress test units being removed from test prior to failure; the standard control chart process needs to be modified. If in addition, the underlying distribution of the measured quantity is not normal, then calculated capability indices based on the control chart will be only approximate.

This paper surveyed techniques which have been available but have seen minimal application to the control charting of data constrained by censoring, particularly due to the detection limit (LOD) situation. The issue of non-normal distributions was also addressed. The basic requirement is the ability to estimate the shape and location of the process under study. The methods of probability/hazard plotting and maximum likelihood estimation will be demonstrated for addressing the problem of measurement values below the LOD. It is assumed that the data are valid and obtained from a properly calibrated instrument. Other issues to be discussed include experimental methods for determining the limit of detection, minimum detectability, minimum concentration, and minimum quality detections.

---

### Total automation of analytical instruments

*Timothy S. Goodfellow, CompuChem Laboratories (3308 Chapel Hill/Nelson Highway, Research Triangle Park, NC 27709)*

To many people, automation of analytical instruments consists of using the instrument with an autosampler. However, the areas of autosampler set-up, validation of results, and the posting of results to a computer database must be addressed before total automation is achieved.

Total automation of instruments was the goal of a project at CompuChem Laboratories, a high volume laboratory specializing in environmental analysis. The results show that total automation is achievable and can provide improvements in quality and productivity.

Autosampler set-up was reduced by 80% through computer programs that automatically create the sample description file needed by the PC that controls the instrument and autosampler. The sample description file is built such that data validation and the posting of results to the database are facilitated. In addition, an autosampler set-up map is produced to assist the technician in putting samples into their correct position in the autosampler rack. Autosampler files and maps are created in advance to allow autosampler racks to be filled while another analytical run is in progress. A post-analysis data verification program is used to ensure that all QC samples are within control limits before being posted to the results database. Manual entry of sample numbers, descriptions and codes during the set-up or posting steps is eliminated.

Real-time control of instrumentation offers the ability to dynamically process samples. Samples requiring dilutions are prepared and reanalysed immediately rather than in a subsequent analytical run. The degree to which dynamic analytical runs, and all automation in general, are possible depends upon the flexibility of control allowed by the instrument manufacturer. Consequently, each instrument will thoroughly test the creativity, resourcefulness, and patience of the automation specialist.

Perhaps the greatest benefit is that analytical runs can become shorter because the reduced overhead of autosampler set-up, data validation, and posting of results no longer requires long analytical runs to offset the high set-up time. Shorter analytical runs offer the strategic advantages to a production analytical facility. Increased throughputs were found to reduce the time required for putting the client's sample data package together.

---

### Integrated networking in multi-system multi-vendor laboratory environments

*Kannan Pashupathy and Dan Holmes (Hewlett-Packard Company, 1601 California Avenue, Palo Alto, CA 94304)*

One of the many reasons for the introduction of networking into an analytical laboratory is the productivity increases that are realized. These could simply be the versatility of networking in moving data from one system to any other networked system, or the ability of end users to remotely access such data and share resources like peripherals over the network.

With the rapid increase in computation power of these analytical data systems, there has been a proliferation of different operating environments, networking protocols, and application user-interfaces. This has led to an increase in the amount of data and often a decrease in the amount of useful information that is available easily and quickly. Lack of data migration paths from older systems to newer ones, and growing re-training needs due to the complexity of today's systems have often frustrated laboratories looking for effective networking solutions today.

Using industry-standard networking based on IEEE 802.3 and ARPA Services, graphical interfaces derived from de-facto standards like Microsoft Windows and X-Windows, efforts at HP have resulted in a software product which is a planned approach to designing and integrating instruments and computing systems either into a single laboratory environment or throughout the enterprise. The overall objective is to provide information access to the entire environment without users having to know or understand the underlying networking concepts or compatibility issues that arise out of the use of dissimilar instruments and data systems.

This paper described the design and implementation strategy for achieving integrated networking across data systems running on DOS-based PCs, Unix-based workstations, real-time operating environments like HP's RTE, and HP Pascal workstations.

---

### The concentration of water soluble volatile organic compounds in water by distillation

*Mark L. Bruce, Richard P. Lee, Marvin W. Stephens (Wadsworth/ALERT Laboratories, Inc., 4101 Shuffel Dr. N.W., North Canton, OH 44720)*

Under the US land disposal restriction (40 CFR part 268.41) for spent solvents, the treatment standard for methanol is 0.25 mg/l for wastewaters containing spent solvents using zero headspace extraction (ZHE). The treatment standard for methanol for all other spent solvent wastes in the waste extract using ZHE is 0.75 mg/l. This paper presented the development of an aqueous sample concentration and cleanup method. When the concentrate is analysed by gas chromatography (flame ionization detector) these regulatory treatment standard levels for methanol are readily achieved. Other water soluble volatile compounds such as acetonitrile, acrolien, acrylonitrile, 1-butanol, 2-butanone, 1,4-dioxane, ethanol, ethyl acetate, 2-methyl-1-propanol, 1-propanol, 2-propanol, propionitrile, have also been concentrated with this method. Distillation time was 5–10 minutes. The concentration factors ranged from 8 to 100 depending on condenser and fractionation column design. The distillation requires 10 to 40 ml of sample. The method detection limits were in the low μg/l range. Two aqueous matrixes have been studied, ground water and the Zero Headspace Extract (an acetate buffer solution).

The first prototype was based on a modified Nielson-Kryger condenser. Concentration factors up to 8 have been achieved with such systems. Other miniaturized condenser designs have improved the concentration factor to 15. A simple glass bead packed fractionation column coupled to a 1/16 inch O.D. Teflon® tube (air condenser) has produced concentration factors above 50.

### A common data and control interface for analytical chemistry instrumentation

*Gary W. Kramer and H. M. Kingston (National Institute of Standards and Technology, Center for Analytical Chemistry, Chemistry Building 222, M/S A-343, Gaithersburg, MD 20899)*

At the National Institute of Standards and Technology Center for Analytical Chemistry, a group of interested parties has been gathered from the private sector and other government agencies to form the Consortium on Automated Analytical Laboratory Systems (CAALS). This Consortium's goal is to foster the creation of automated chemical analysis systems which will improve the quality of analytical data, facilitate the entire analytical process, and promote the standardization and transfer of analytical methods, while providing industry with competitive advantages in chemical measurement technology.

Currently, most of the technology necessary to create systems for automating entire chemical analyses exists. However, interconnecting today's instruments to build such systems is often a difficult process. Many obstacles stem from a lack of general guidelines and standards in critical areas of data and control information interchange. As one of its initial objectives, CAALS has undertaken the task of defining and promoting a common control and data interface standard for analytical instruments. Application of such standardized communications methods along with a modular workcell concept can dramatically ease the task of fabricating automated analysis systems. Once constructed, automated chemical analysis systems will prove to be a valuable tool for achieving and maintaining quality assurance in the laboratory.

### General purpose robotic HPLC sample processor

*L. J. Lorenz, D. E. Reed, R. J. Lykins, J. L. Bollinger and J. Zynger (Lilly Research Laboratories, Eli Lilly and Company, Lilly Corporate Center, Idianapolis, IN 46285)*

Laboratory robotics has generally followed a course where a robotic system has been designed to mimic a previously developed manual procedure. This presents a problem since most procedures vary. Therefore, a different system is needed for each application. This type of approach requires a large front-end development effort which is lost as the application changes or ceases to exist.

An alternative approach to the utilization of laboratory robotics requires the development of a general purpose robotic system. Applications are then developed to fit the robotic system. Such an approach allows for robotic systems which can handle a large number of different methods without alteration of the generic robotic system.

Such a system has been developed as a sample processor for HPLC. Today, many samples which have very limited solution stability are examined by HPLC. This poor solution stability limits the ability to use classical automated autoinjectors for servicing an HPLC instrument. A robotic system can overcome this problem by preparing the samples just prior to analysis.

The robotic system is designed to make use of commercially available devices wherever possible. The system will handle liquid samples when desired but is primarily designed to handle dry powders. The system will process a dry sample which is presented preweighed in a test tube. A solvent is added to the tube and the material is put in solution through a series of shaking and steps. The sample can undergo a subsequent dilution step with mixing if desired. The solution is then transferred to a syringe filter which filters the sample and also acts as the driving force to push the sample through selection valves to direct the sample to a specific instrument and finally through a sampling valve to inject the sample into the HPLC system. The robotic system incorporates appropriate relays and logic to start an HPLC gradient, to autozero detectors, and to start data collection for the sample. The robot documents all key steps in the process and documents all error recovery attempts when a problem is detected.

